# Analysis of the nonlinear dynamic response of guide rails for a suspended buffer

**DOI:** 10.1371/journal.pone.0210185

**Published:** 2019-01-17

**Authors:** Yang Tai, Shuai Guo, Baifu An, Feng Ju

**Affiliations:** 1 School of Mines, China University of Mining and Technology, Xuzhou, China; 2 Key Laboratory of Deep Coal Resource Mining, China University of Mining and Technology, Xuzhou, China; 3 State Key Laboratory for GeoMechanics and Deep Underground Engineering, China University of Mining and Technology, Xuzhou, China; Central South University, CHINA

## Abstract

Guide rails’ nonlinear dynamic response to the collision process between gangues and the suspended buffer is the basis for its intensity examination and fatigue prediction. This paper established a numerical model for simulating the dynamic response of buffer’s guide rails using Pro/E and Femap based on an analysis of the basic structural components of the suspended buffer. The effects of the gangues diameter and feeding rate on the guide rails’ dynamic response were investigated, which were mainly revealed by a tension in guide rails. Simulation results showed that the tension in guide rails rapidly increased to a peak value after the suspended buffer was impacted by gangues, followed by a periodic vibration. The peak tension, maximum and minimum tensions within the periodic vibration were exponential functions of the gangues diameter. And they depended linearly on the feeding rate. The vibration frequency of the tension in guide rails was an exponential function of the gangues diameter but did not depend on the feeding rate. Based on the backfilling workface parameters used in D.Ping coal miner’s 15601 working panel, the feeding rate of 500 t/h was selected. The diameter of the crushed gangues was selected to be 50 mm based on the strength of guide rails. Their expected service life is 54.2 years. Finally, an industrial test was performed in D.Ping coal miner’s 15601 working panel and the guide rails operated steadily. The measured vibration parameters of the tension in guide rails agree well with that of numerical predictions, which verified the reliability of the numerical model to some extent.

## 1 Introduction

**Fig 1:**
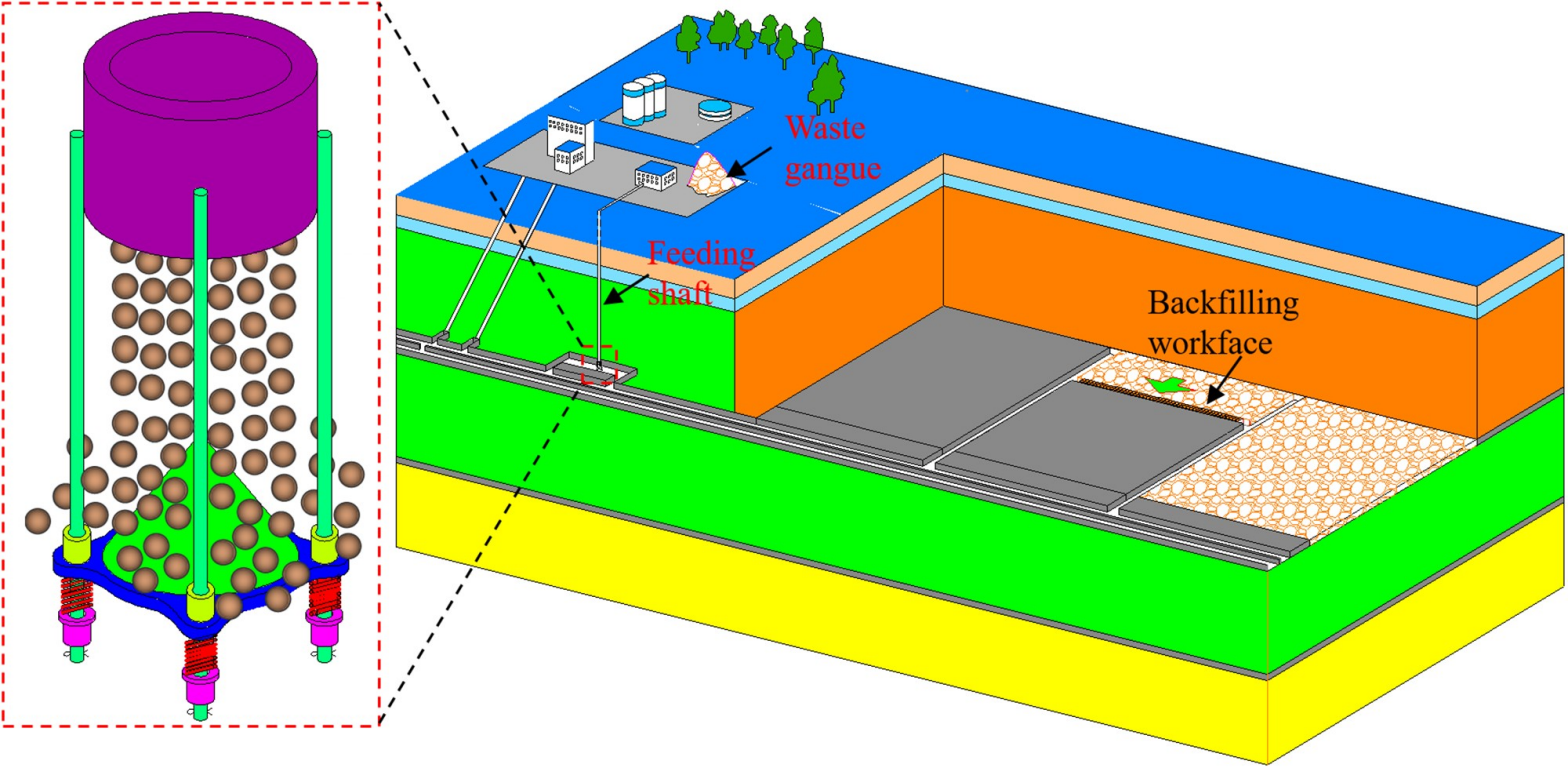
Fully mechanized mining system and the suspended buffer.

Fully mechanized mining with the solid backfilling technology is one of the solutions to the “three-underground” coal mining problems [1] [2] [3]. Gangue is widely used as the backfilling material [4] [5], which not only significantly reduces the backfilling cost, but can also solve the pollution problem induced by piling up gangues on a ground surface. However, the underground gangues storage silo may be damaged by gangues with high speed if they are transported directly from surface to a feeding shaft bottom [6]. Therefore, the safe and efficient transport of gangues from a surface to a feeding shaft bottom has become the crucial point in the development of the fully mechanized mining with the solid backfilling technology. To solve this problem, a suspended buffer has been designed after years of research, which is shown in Fig 1. As the core equipment in the gangues backfilling system, the suspended buffer reduces the speed of gangues coming from the surface to a reasonable value and therefore prevent damage to the storage silo [7] [8]. The suspended buffer is fixed at the feeding pipe by guide rails, which not only provide tension for the buffer but also help to avoid its horizontal swing. The dynamic response of guide rails under collision process determines its intensity check and fatigue prediction, which are significant for the safety and service life of the suspended buffer. The dynamic response of guide rails is affected by the gangues diameter as well as the feeding rate, the effects of which require detailed investigation and analysis.

Domestic and foreign scholars have done a lot of research on the motion state of feeding materials, collision characteristics between feeding material and buffer, buffer material selection and buffer structure design. Regarding motion state of feeding materials, Zhang *et al*. [9] revealed that the effects of particle size and density on the motion state of gangue particles in the feeding pipe. In terms of collision characteristics between feeding material and buffer, Liu *et al*. [10] analyzed the buffer’s structural stability and the characteristics of its response to vibration. Ju *et al* [6] investigated the characteristics of both elastic and plastic collisions between gangues and a buffer within the collision cycle, adopting a theoretical analysis method. The buffer spring vibration equation and the dynamic deflection equation of the supporting beam were derived based on the analysis results. In respect of buffer material selection, Huang *et al*. [11] investigated the ultimate bearing capacity of foam buffer package materials under static and dynamic loadings. They adopted highly micronized fibers to increase the compressive and tensile strength of the foam buffer package. Wen *et al*. [12] studied the EPS buffer layer of motorcycle helmets and experimentally obtained its energy absorption characteristics with different buffer densities. In terms of buffer structure design, Wang *et al*. [13] proposed a honeycomb buffer and investigated its energy absorption efficiency varying buffer sizes at impact test. Uddin *et al*. [14] designed a buffer with hexagonal hollow tubes and the buffer has high energy absorption efficiency to control high-speed falling equipment.

The aforementioned studies of buffers have been focused on motion state of feeding materials, collision characteristics between feeding material and buffer, buffer material selection and buffer structure design. Regarding motion state of feeding materials, while an in-depth study on the dynamic response of guide rails of suspended buffer is still missing. Such a study can provide a basis for intensity check and fatigue prediction of guide rails, which is necessary to ensure their safety and lifetime. In this paper, it was investigated that the effects of gangues diameter and feeding rate on the dynamic response of guide rails by numerical simulation based on the analysis of structural components of the suspended buffer. Then, the intensity check and fatigue prediction were performed for guide rails. The analysis results were used to determine the feeding rate in D.Ping coal mine based on the backfilling workface parameters applied there. We estimated the appropriate gangues diameter and service life of guide rails. Finally, the industrial test was performed in D.Ping coal mine.

## 2 Structural components of a suspended buffer and design principles of guide rails

### 2.1 Structural components of a suspended buffer

**Fig 2:**
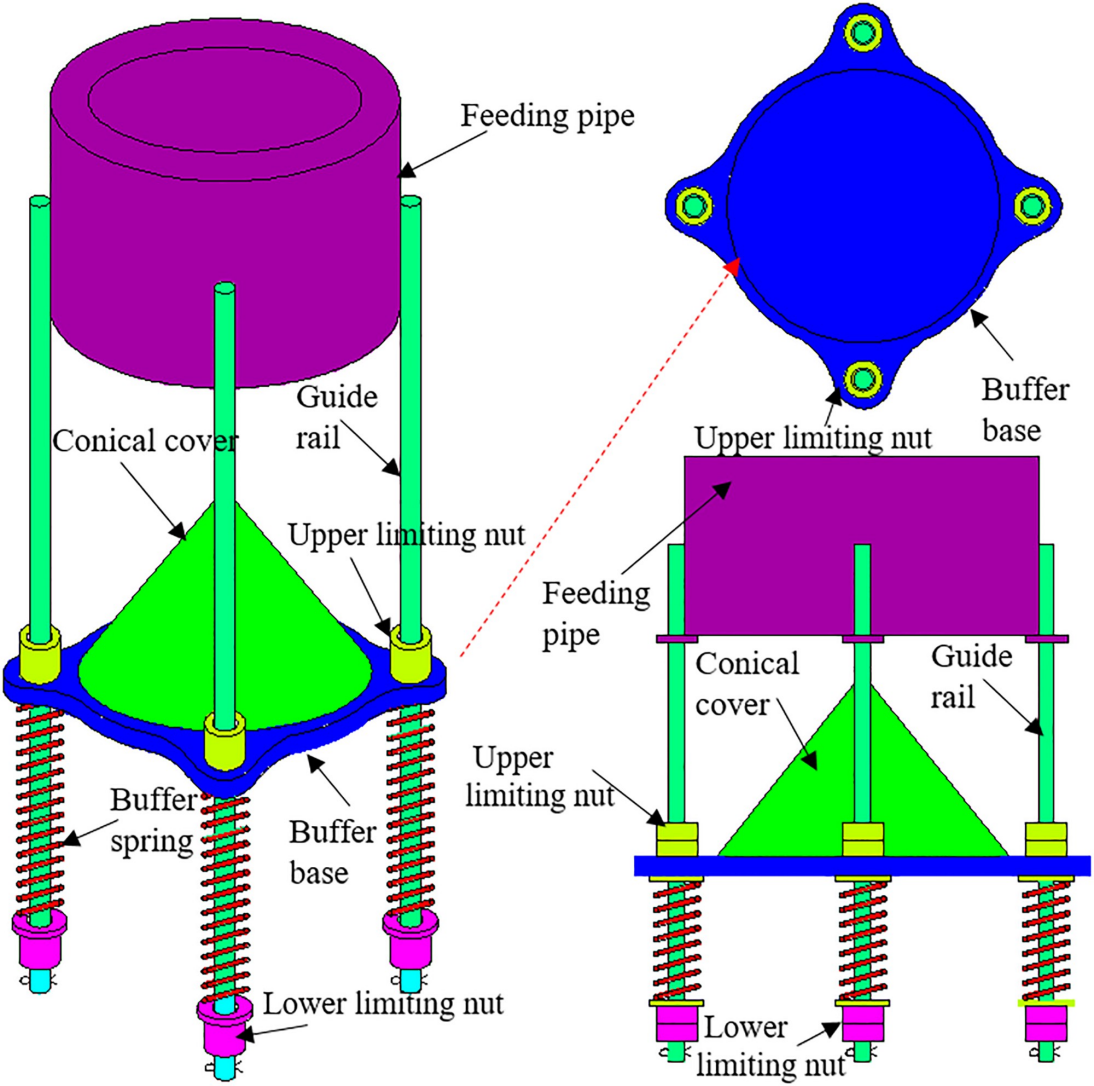
Structural components of the suspended buffer.

As shown in Fig 2, the suspended buffer is mainly composed of a buffer mechanism and a limiting mechanism. The buffer mechanism includes a conical cover, a buffer base, and buffer springs, while the limiting mechanism includes the upper and lower limiting nuts as well as guide rails. The conical cover is fixed onto the buffer base, which is sleeve-connected with the guide rails. Upper ends of the buffer springs are connected to the buffer base, while their lower ends are fixed to the lower limiting nuts. Both the upper and lower limiting nuts are connected or fixed to the guide rails, the upper ends of which are fixed at the outer wall of the feeding pipe by welding. Gangues are fed to the buffer device through the feeding pipe, which, after being retarded by the conical cover, scattered to a storage silo at a low speed. The buffer mechanism achieves repeated buffering and recovery under the combined action of the buffer springs and the gangues.

### 2.2 Design principles of guide rails

The guide rails are the components that fix the suspended buffer at the feeding pipe. Also, they fix the vibration direction of buffer springs. As gangues collide with the buffer, the guide rails are pulled and start to vibrate. Therefore, the guide rails are required to have certain tensile strength. On the other hand, the guide rails experience a long-term cyclic loading, which may lead to the formation of cracks and eventually the fatigue failure. As a result, the guide rails are also required to have the certain resistance to fatigue. We selected Q345 steel grade or above for the guide rails [10] [15]. Four guide rails were used for the stability of the suspended buffer.

## 3 Numerical model for the dynamic response of suspended buffer’s guide rails

In order to analyze the effects of gangues diameter and feeding rate on the dynamic response of suspended buffer’s guide rails, the out-of-pipe speed for gangues was approximated first. Then a numerical model for simulating the dynamic response of the rails was established. After calibration of the material parameters of gangues, the modeling scheme was determined.

### 3.1 Out-out-pipe speed for gangues

The forces on gangues in the feeding pipe include gravity, buoyant force, added mass force and air resistance. Zhang *et al*. [9] deduced an expression for the speed *u* of gangues in the feeding pipe:

$$u=f+\frac{\sqrt{c}\;\text{tan}\left[\frac{\sqrt{b}\sqrt{c}t}{a}-\text{arctan}(\frac{\sqrt{b}f}{\sqrt{c}})\right]}{\sqrt{b}}$$ (1)

where $a=\rho_{p}\frac{\pi D^{3}}{6}$, $b=\frac{\pi D^{3}}{8}\rho_{f}C_{D}$, $c=\rho_{p}\frac{\pi D^{3}}{6}g$ and *f* = *u*_*f*_. In the above equation, *D* is the gangues diameter, *ρ*_*p*_ = 2500 kg/m^3^ is the gangues density and *g* = 9.8/*ms*^-2^ is the gravitational acceleration. *C*_*D*_ is the drag coefficient, the value of which is usually taken as 0,44. Finally, the air in the feeding pipe has a density *ρ*_*f*_ = 1.205 Kg/m^3^ and an average speed is *u*_*f*_ = 2m/s.

El-Behery *et al*. [16] [17] [18] concluded that wasted gangues, subjected to various types of loading, eventually accelerated to a peak and constant speed, at which the impact load on the suspended buffer was the largest. As a conservative estimation, the peak speed was adopted as the out-of-pipe speed of gangues. The time at which *u* reaches the peak value can be obtained when *du* = 0, which leads to

$$1-{\left\{\text{tan}\left[\frac{\sqrt{b}\sqrt{c}t}{a}-\text{arctan}(\frac{\sqrt{b}f}{\sqrt{c}})\right]\right\}}^{2}=0$$ (2)

Where parameters *a*, *b* and *c* in Eq (2) are same with that in Eq (1), which are relative with gangues diameters *D*.

Substituting gangues diameters *D* = 20, 40, 60, 80 and 100 mm into Eq (2), one can obtain that for these gangues the times at which they reach their maximum speeds are 8.6, 12.1, 14.8, 17.1 and 19.0 seconds, respectively. Then, the maximum speeds can be calculated using Eq (1), leading to *v* = 33.0, 47.6, 58.7, 68.1 and 76.4 m/s, respectively.

### 3.2 Model establishment

In this work, the Pro/E 3D modeling software was adopted to construct the solid model of the suspended buffer, in which commands such as stretch, rotate and mirror have been used. The generated 3D model was then imported into Femap using IGS as an intermediate file. Finally, meshing, element selection, boundary conditions and material properties assignments for the solid model were performed in Femap.

**Fig 3:**
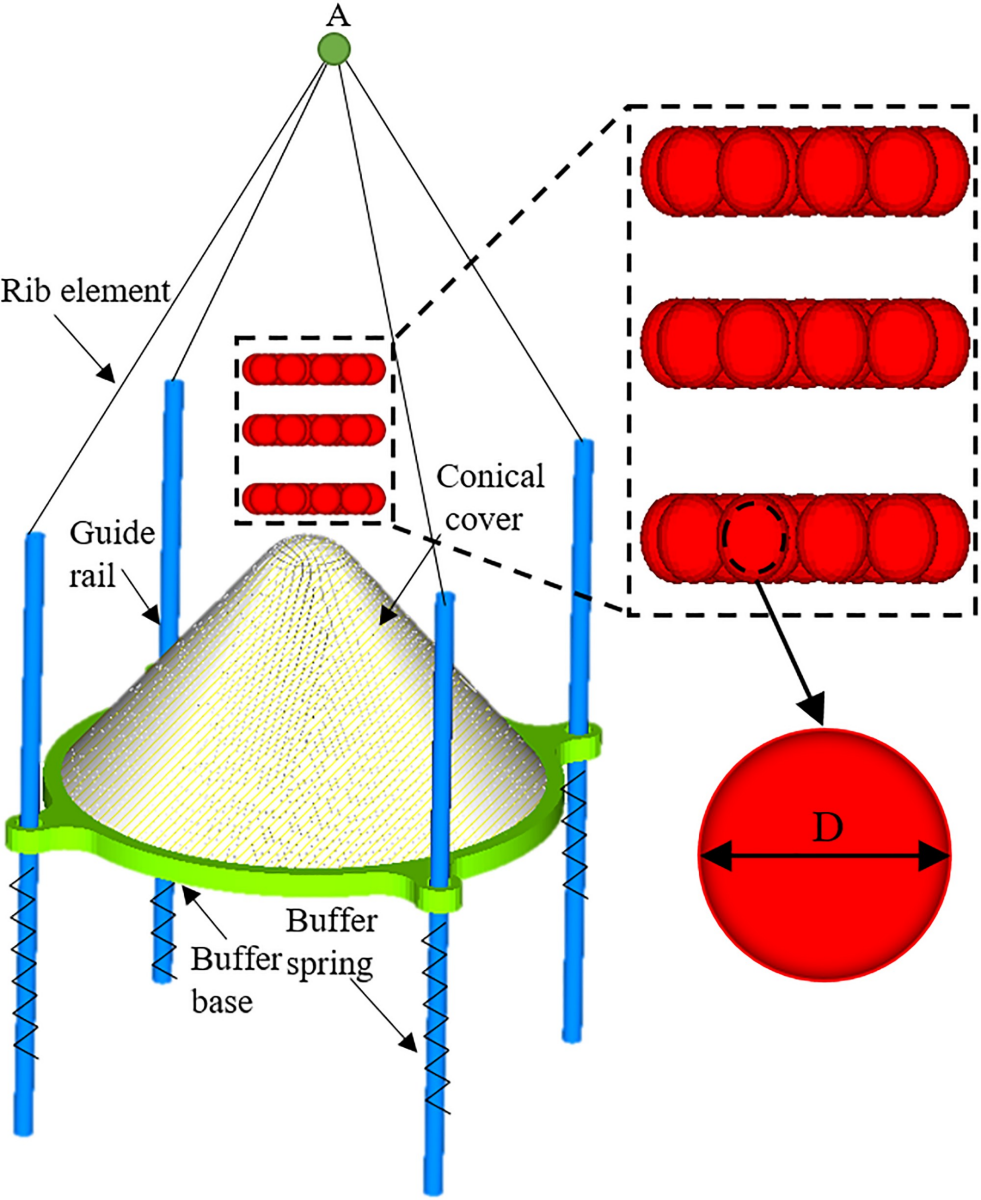
Finite element model.

As shown in Fig 3, conical cover and buffer base were discretized using quadratic shell elements, while buffer springs and guide rails were discretized using the DOF spring elements and the quadratic beam elements, respectively [19]. A solid element was selected for gangues [20]. The tops of guide rails and bottoms of buffer springs overlapped with each other, while the top ends of guide rails were connected to point A via rib elements [21]. Also, the multi-point constraint was adopted to couple six directional degrees of freedom between springs and the buffer base. The conical cover and buffer base were modeled using the bilinear isotropic hardening model with elastic modulus [22] [23] *E*_1_ = 210 GPa, Poisson’s ratio *μ*_1_ = 0.30 and tangent modulus *T*_1_ = 21 GPa. Gangues were assumed to be spherical and governed by the Mohr-Coulomb yielding criteria [24] [25]. The elastic modulus and Poisson’s ratio for waste particles are *E*_2_ = 15.0 GPa and *μ*_2_ = 0.27, respectively. Also, it has a cohesive force *c*_2_ of 6.2 MPa and an internal friction angle *φ*_2_ of 23°. The four buffer springs between the upper and lower limiting nuts had a stiffness 3.2×10^5^ N/m. Their roles as damping materials are reflected through structural damping [26] [27]:

C=αM+βK (3)

where *C* is the damping matrix, *M* is the mass matrix and *K* is the stiffness matrix. *α* and *β* are the mass and stiffness damping ratio coefficients, respectively. Based on theoretical analysis and trial calculation, *α* = 0.23 and *β* = 0 were adopted in this work [26, 28]. So damping matrix *C* can be expressed as *C* = 0.23*M*.

### 3.3 Parameters calibration for gangues

In this model, the material properties for conical cover, buffer base, and guide rails were obtained from experimental measurements. However, as gangues were simplified as spheres, the direct use of their properties measured in a lab would inevitably lead to simulation error. It was, therefore, necessary to calibrate the material properties for gangues. This was achieved by permanent adjusting its elastic modulus *E*_2_, Poisson’s ratio *μ*_2_, cohesive force *c*_2_ and internal friction angle *φ*_2_, such that the vertical vibrational displacements of the springs in simulation agreed with that of field measurements.

#### 3.3.1 Obtaining gangues

**Fig 4:**
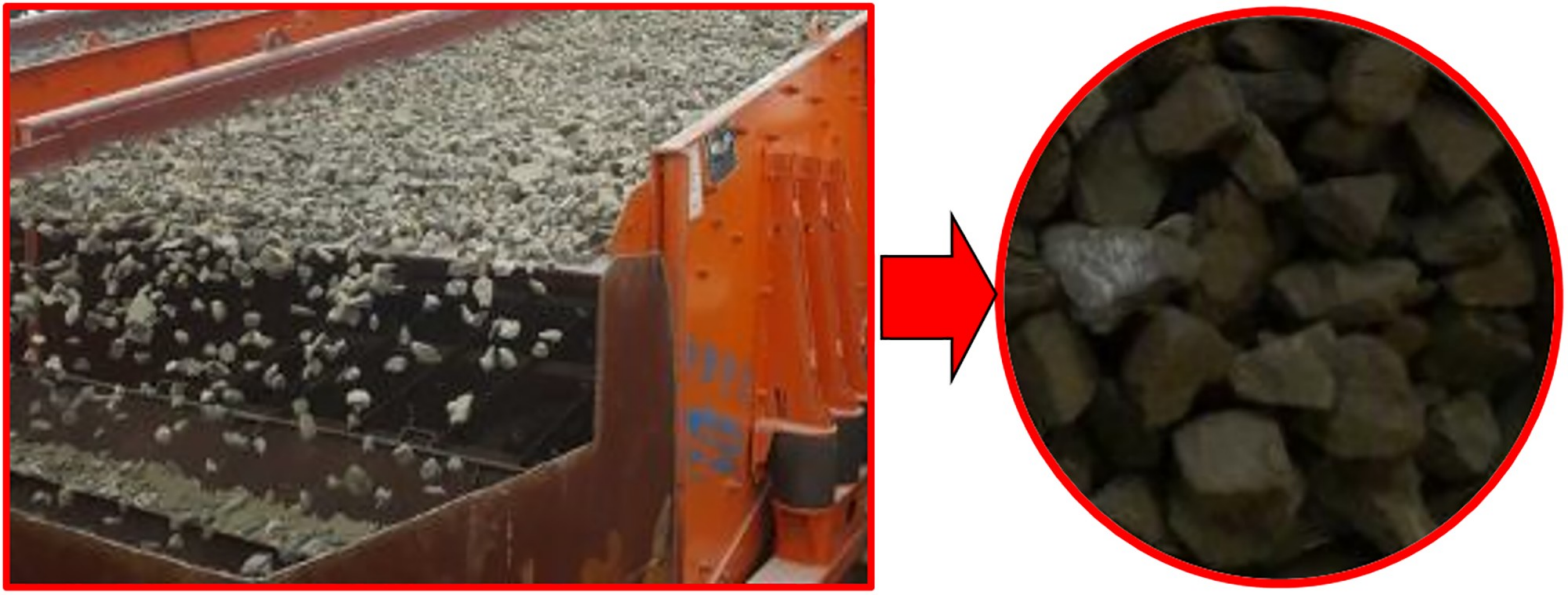
Screening process of gangues.

Gangues with certain diameter must be obtained to calibrate their material parameters. This was achieved using the multistage vibrating screening as shown in Fig 4, which screened out gangues with an average diameter of 40 mm. The screened gangues were then fed into the feeding pipe. After coming out from the pipe, gangues collided with the suspended buffer.

#### 3.3.2 Parameters calibration

Monitoring the vertical displacements of buffer springs is relatively simple. Therefore, a YWD-80 displacement sensor was installed for the real-time monitoring of its vertical displacement. The maximum data acquisition frequency of this device can reach up to 100 kHz. The device was fixed near the spacing hole. The monitoring time was 5 s, during which the data were obtained and stored.

**Fig 5:**
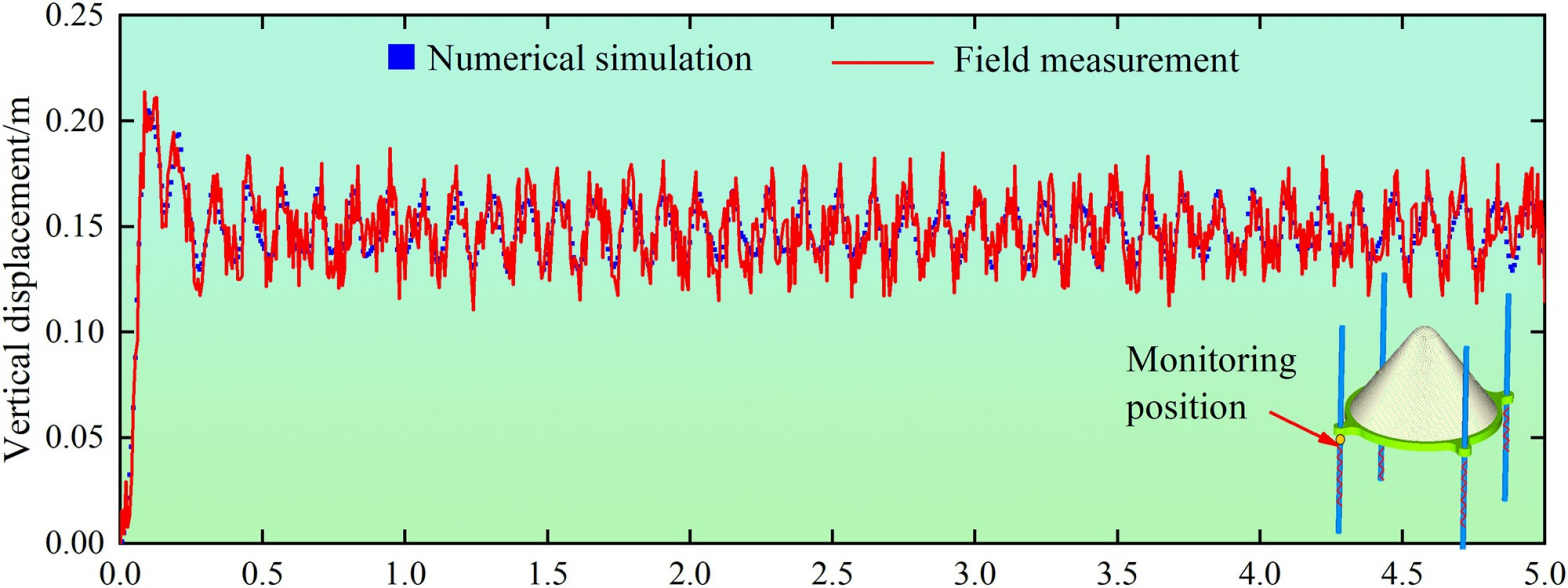
Vertical displacement of the spring in numerical simulation and field measurement.

The vertical vibrational displacement of the buffer springs for *D* = 40 mm was obtained by the YWD-80 sensor measurements and the data processing system. By permanent adjustment of the parameters for gangues, the vertical displacement in the numerical simulation was converged to that of the field measurement, as shown in Fig 5. The resulting gangue parameters were *E*_2_ = 16.1 GPa, *μ*_2_ = 0.28, *c*_2_ = 5.6 MPa and *φ*_2_ = 22°.

Fig 5 shows that for *D* = 40 mm, displacements of the springs in numerical simulation and field measurement were 0.214 m and 0.205 m, respectively, with the corresponding vibration periods of 0.169 s and 0.173 s. Both the magnitude and trend of the vertical displacement agreed well with the field measurement, which to some extent verified the appropriate selections of gangues parameters.

### 3.4 Research schemes

The way to fixed guide rail and material of guide rail is an effective way to solve the cost of buffer and ensure the safety of buffer. However, the choice and design of the both need to know the dynamic response of guide rails. So the paper researches on the effect of gangue diameter and gangue feeding speed on the guide rails’ dynamic response. Two research schemes was designed to investigate the effects of gangues diameter and feeding rate on the dynamic response of suspended buffer’s guide rails. For the gangues diameter, it is generally 0~100 mm after washing. And according to the backfilling capacity of workface, the gangue feeding speed is 100~700 t/h. Scheme I fixed the feeding rate at 500 t/h and the effect of the gangues size was investigated by varying the diameter from 20 to 100 mm with a step size of 20 mm. In contrast, Scheme II fixed the gangues diameter at 60 mm and the effect of the feeding rate was investigated by varying it from 100 to 700 t/h with a step size of 200 t/h. Details of the research schemes are provided in Table 1.

**Table 1:** Modeling schemes.

Schemes	Gangues diameter /mm	Out-out-pipe speed /m s^-1^	Feeding rate /t/h
I	20	33.0	500
	40	47.6	
	60	58.7	
	80	68.1	
	100	76.4	
II	60	58.7	100
			300
			500
			700

## 4. Analysis of influence factors of the guide rail’s dynamic response

The dynamic response of the suspended buffer’s guide rails to the impact load delivered by gangues is particularly revealed by a variation of tension in the rails. Therefore, this paper investigates the effects of the gangues diameter and feeding rate on the dynamic response of the rails by monitoring the vertical force at point A in the numerical simulation, which can be used to approximate tension in the guide rails.

### 4.1 Gangues diameter

**Fig 6:**
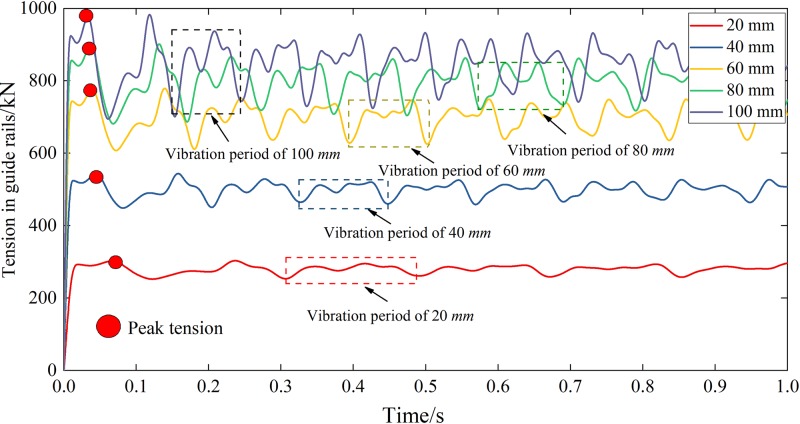
Effects of the gangues diameter on the tension in the guide rails.

The gangues generally have a relatively large diameter, so they are usually required some crushing before they can be used as a backfilling material. The diameter of the crushed gangues is directly related to the technology and cost of crushing, with a more complex technology and a higher cost of smaller crushed gangues. However, a smaller diameter gangue reduces the out-of-pipe speed and thus reduces the impact of gangues on the buffer, leading to a smaller tension in guide rails. Therefore, it is of great significance to determine the critical diameter of crushed gangues. Fig 6 summarizes the simulation results for Scheme I, which will be used to study the effect of the gangues diameter on the dynamic response of the guide rails.

One can conclude from Fig 6 that:

1. Under the impact of gangues, the peak tension in guide rails increased rapidly. For *D* = 20, 40, 60, 80 and 100 mm, the corresponding peak tension values were 303.6, 544.0, 747.5, 901.6 and 983.2 kN, which occurred at 0.070, 0.047, 0.041, 0.039 and 0.034 s, respectively.
2. After reaching the peak tension, the tension in the guide rails began to vibrate periodically. For *D* = 20, 40, 60, 80 and 100 mm, the tensile vibration periods of the guide rails were 0.180, 0.120, 0.106, 0.010 and 0.090 s, corresponding to frequencies of 5.556, 8.333, 9.434, 10.000 and 11.111 Hz, respectively.
3. After entering the periodic vibration, the maximum tensions in the guide rails for *D* = 20, 40, 60, 80 and 100 mm were 287.2, 514.1, 754.4, 870.1 and 937.7 kN, while the minimum tensions were 257.0, 454.9, 637.8, 709.2 and 729.2 kN respectively.

**Fig 7:**
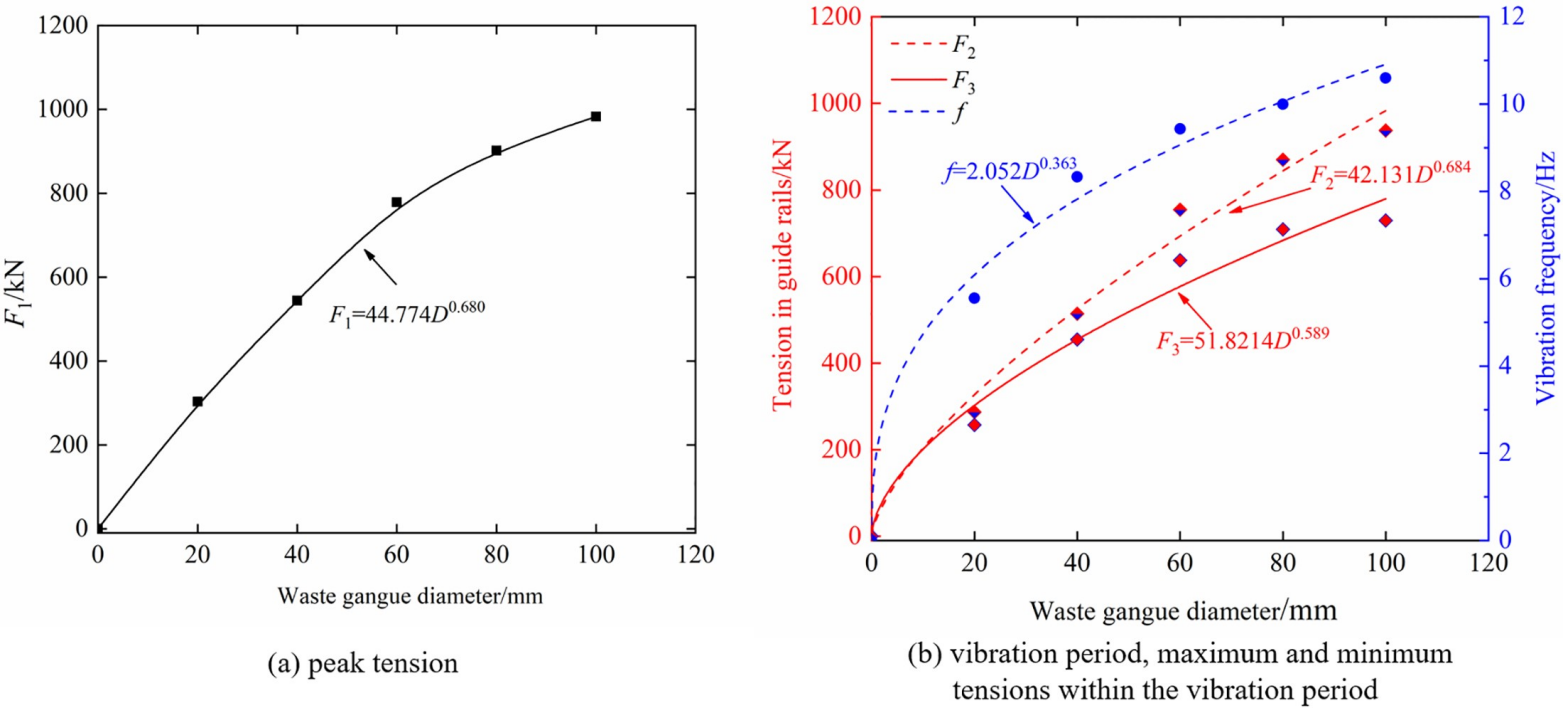
The effect of gangue diameter on vibration parameters of tension in the guide rails (a) peak tension; and (b) vibration period, maximum and minimum tensions within the vibration period.

In order to obtain expressions for the peak tension *F*_1_, vibration frequency *f*, maximum tension *F*_2_, and minimum tension *F*_3_ within the vibration period in guide rails as functions of gangues diameter, variations of these parameters versus *D* were plotted in Fig 7. Fitting the curves using *Origin* commercial software yielded the following relations: *F*_1_ = 44.776*D*^0.678^, *f* = 2.052*D*^0.363^, *F*_2_ = 42.131*D*^0.674^, and *F*_3_ = 51.821*D*^0.589^.

### 4.2 Feeding rate

**Fig 8:**
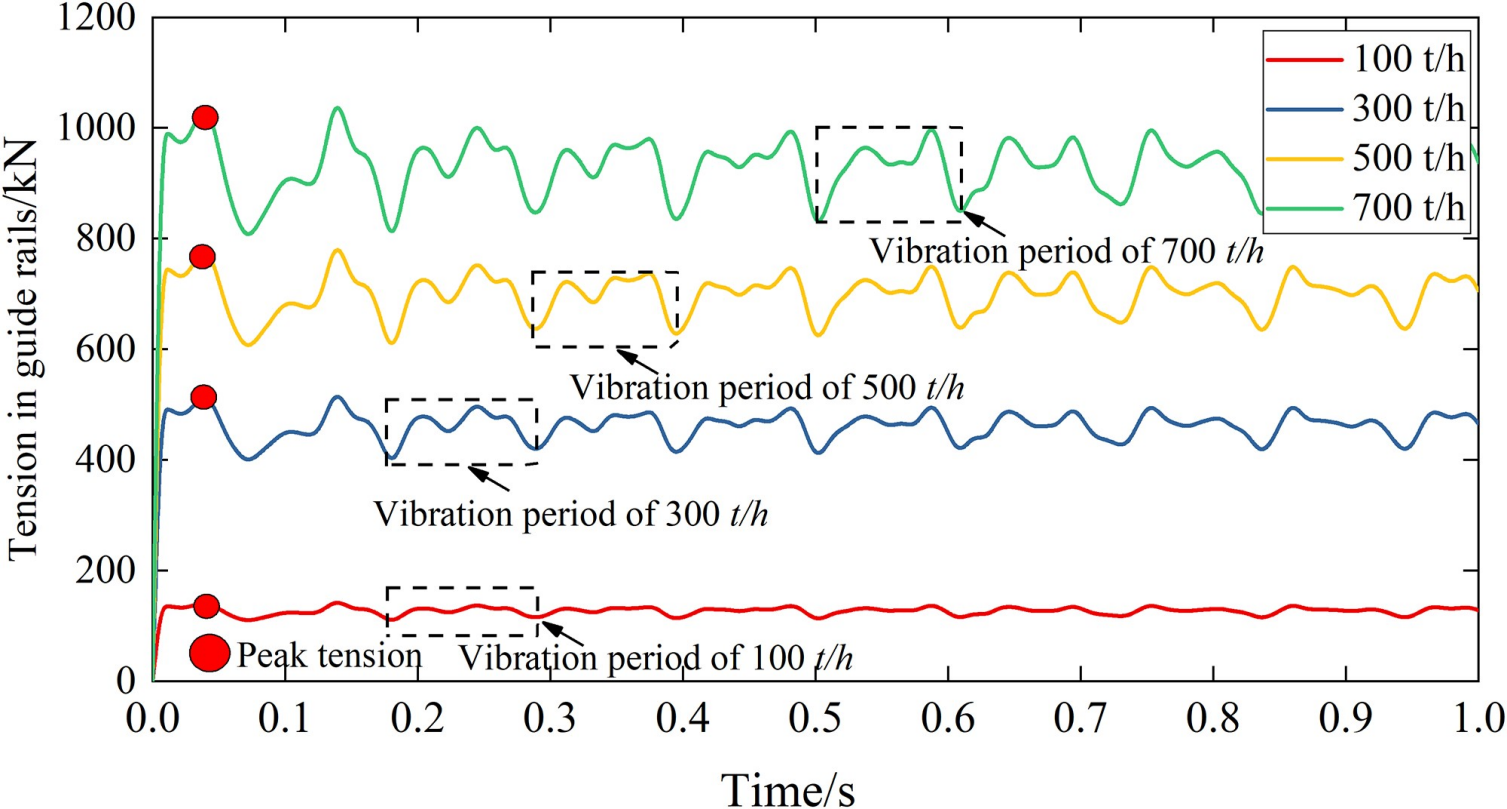
Effect of the feeding rate on the tension in the guide rails.

The feeding rate is determined by the backfilling speed of gangues in a backfilling workface, which is limited by the parameters of the backfilling workface such as the working panel length, a height of the filling, and the mining rate, etc. Variations of these parameters would require an appropriate adjustment of the feeding rate. Fig 8 summarizes the simulation results for Scheme II which will be used to investigate the effect of the feeding rate on the tension in the guide rails.

One can conclude from Fig 8 that:

1. The peak tension in guide rails increased with the increase of the feeding rate. For *v* = 100, 300, 500 and 700 t/h, the corresponding peak tension *F*_1_ values were 136.0, 493.3, 747.5 and 994.2 kN, respectively, all of which occurred at *t* = 0.047 s.
2. After reaching the peak tension, the guide rails began to vibrate periodically. The vibration periods for all the cases simulated in Scheme II were 0.120 s, corresponding to a frequency of 8.333 Hz.
3. After entering the periodic vibration, the maximum tensions within the vibration period in guide rails for *v* = 100, 300, 500 and 700 t/h were 136.9, 517.9, 751.0 and 986.1 kN, while the minimum tensions were 112.3, 408.4, 618.9, and 827.8 kN, respectively.

**Fig 9:**
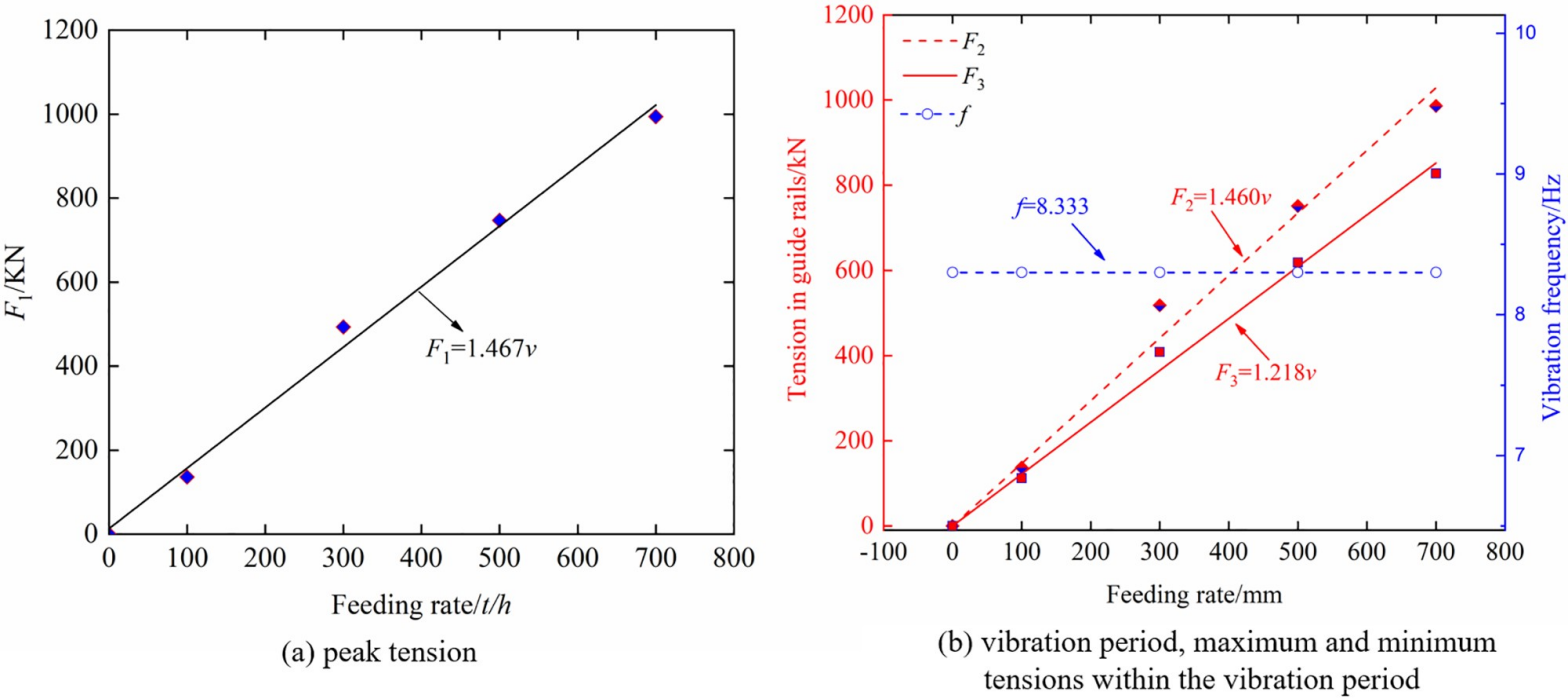
The effect of the feeding rate on vibration parameters of tension in the guide rails (a) peak tension; and (b) vibration period, maximum and minimum tensions within the vibration period.

In order to obtain expressions for the peak tension *F*_1_, vibration frequency *f*, maximum tension *F*_2_, and minimum tension *F*_3_ within the vibration period in the guide rails as functions of the feeding rate *v*, variations of these parameters versus *v* were plotted in Fig 9. Fitting these curves using *Origin* commercial software yielded the following relations: *F*_1_ = 1.467*v*, *f* = 8.333, *F*_2_ = 1.460*v* and *F*_3_ = 1.218*v*.

## 5 Analysis of the strength and fatigue of the guide rail

### 5.1 Intensity check

The peak tension *F*_1_ in guide rails is an important parameter for the intensity check. Section 4 shows that *F*_1_ is an exponential function of the gangues diameter and a linear function of the feeding rate, respectively. Fitting the data using *Origin* yielded the following expression for *F*_1_ as a function of *D* and *v*: *F*_1_ = 0.0896*vD*^0.678^. When four guide rails were used for the suspension buffer, *G*, the force on each one, could be expressed as

$$G=0.0224vD^{0.678}$$ (4)

The feeding rate is determined by the amount of the gangues filling on the working panel. Assuming a number, *A* working panels with a length of *L* and the mining height of *H* to be filled by gangues, the feeding rate can be expressed as,

$$v=\frac{LHV\eta A\gamma }{T}$$ (5)

where *V* is the mining speed, *η* is the extra-coefficient of a gangues, *γ* is the unit weight of a gangues and *T* is the effective feeding time, respectively. Substituting Eq (5) into Eq (4) yielded:

$$G=0.0224\frac{LHV\eta A\gamma D^{0.678}}{T}$$ (6)

Then the peak tensile stress *σ*_1_ on each rail for the guide rails with diameter *d* can be expressed as

$$\sigma_{1}=0.0896\frac{LHV\eta A\gamma D^{0.678}}{\pi Td^{2}}$$ (7)

When the allowable stress of guide rails is *σ*_0_, it was drawn:

$$\sigma_{1}<\sigma_{0}$$ (8)

### 5.2 Fatigue prediction

Fatigue prediction of the guide rails is related to the state of tension in the rails during the vibration period. Similar to Section 5.1, fitting the data using *Origin* yielded the expressions for the maximum tension *F*_2_, minimum tension *F*_3_ within the vibration period as well as the vibration frequency *f* as functions of the gangues diameter *D* and feeding rate *v*:

$$F_{2}=0.0843vD^{0.674}$$ (9)

$$F_{3}=0.1036vD^{0.589}$$ (10)

$$f=2.052D^{0.363}$$ (11)

Then, for guide rails with diameter *d*, the maximum tensile stress *σ*_2_ and minimum tensile stress *σ*_3_ on each rail within the vibration period can be expressed as

$$\sigma_{2}=\frac{0.0843vD^{0.674}}{\pi d^{2}}$$ (12)

$$\sigma_{3}=\frac{0.1036vD^{0.589}}{\pi d^{2}}$$ (13)

Wang and Zhao [29, 30] provided the relation curves between the number of cycle *N* and strain amplitude for Q345 material under cyclic loading, which after fitting in *Origin* can be expressed as

$$\varepsilon =1.356\times 1.0^{-5}(1+\frac{4931}{N^{0.299}})$$ (14)

Then the relation between the number of cycle *N* and stress amplitude within the vibration period can be expressed as:

$$S=2.85(1+\frac{4931}{N^{0.299}})$$ (15)

where the stress amplitude within the vibration period *S* is half the difference between the maximum and minimum tensile stresses in the rail within the vibration period, i.e.,

$$S=(\sigma_{2}\text{-}\sigma_{3})/2$$ (16)

To ensure the service life of the buffer base and the conical cover to be at least a year (the mine operates 330 days a year), one can infer from Eq (11) that each year the number of periodic vibration *n* will be

$$n=330\times 4\times 3600f=9751104D^{0.363}$$ (17)

The service life of suspended buffer’ guide rails can be expressed as

t=N/n (18)

## 6 Engineering application

### 6.1 Engineering background

**Fig 10:**
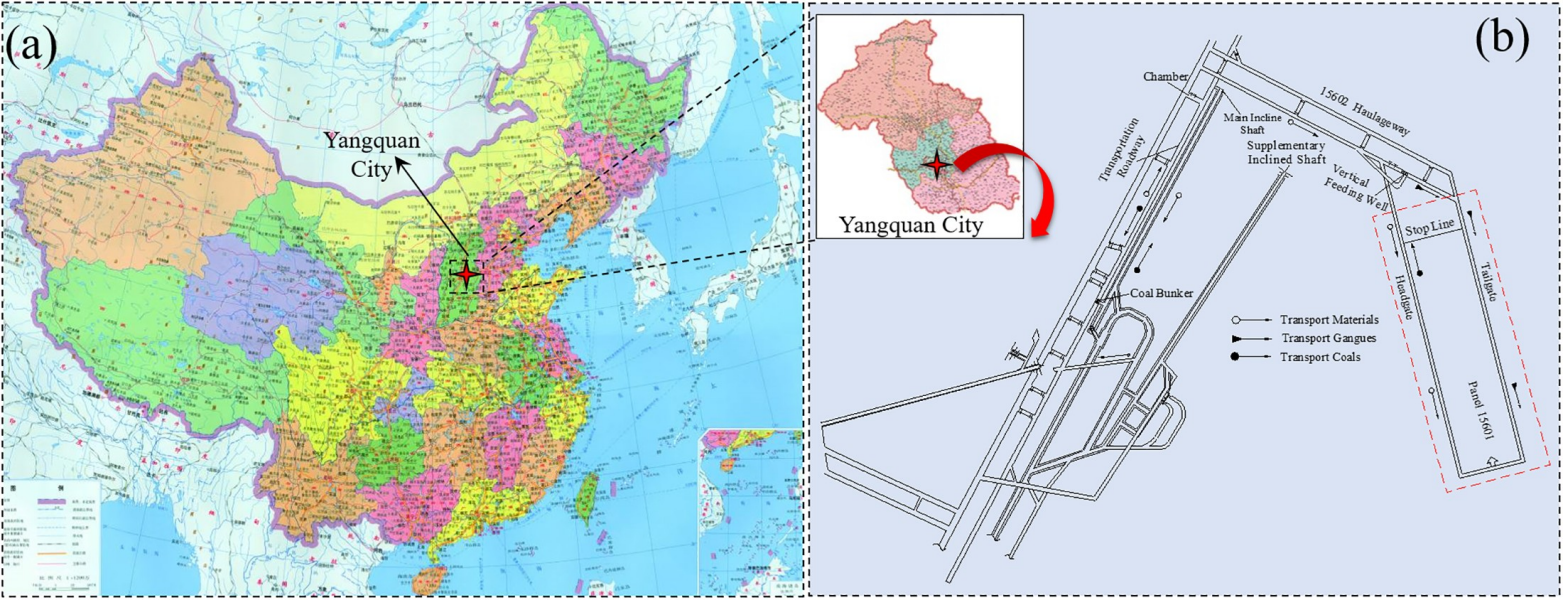
Engineering background (a) location of D.Ping coal mine; and (b) the layout of the working panel.

As shown in Fig 10(a), D.Ping coal mine is located in Yangquan City, Shanxi Province, China, with recoverable reserves of 4.08 million tons. Assuming an annual output of 1.2 million tons, Coal resources in the mine will be exhausted in only 3~5 years. However, there is a large amount of coal that is under the buildings and farmlands within the coal mine area with estimated reserves of 73.54 million tons, 63.24 million tons of which are recoverable. As a result, mining under buildings and farmlands has become the major bottleneck for the sustainable development of D.Ping coal mine. Therefore, as shown in Fig 10(b), it was decided to recover 15601 working panel at D.Ping coal mine using the gangues backfilling technique.

The parameters for the 15601 working panel are summarized in Table 2. Substituting values in Table 2 into Eq (5), one can derive the feeding rate *v* = 504 t/h. For the convenience of equipment selection, *v* = 500 t/h was used.

**Table 2:** The parameters for the15601 working panel.

Workface length (L/m)	Backfilling height (H/m)	Mining speed (*V*/m/d)	Extra coefficient (S)	Unit weight (γ/t/m^3^)	Effective feeding time (T/h)
80	3.0	2.4	1.4	2.5	4

### 6.2 Reasonable particle diameter

The Q345 material was used for the guide rails, which has the yield strength of 345 MPa. Then the allowable stress was 230 MPa for a safety factor of 1.5. The feeding rate in D.Ping coal mine is 500 t/h and the diameter of the guide rails is 30 mm. Substituting these parameters into Eqs (7) and (8) yields

$$\frac{44.776D^{0.678}}{\pi \times 0.03^{2}}<230$$ (19)

which indicates that the gangues diameter *D* should be less than 51.7 mm. We used *D* = 50 mm value for the convenience of equipment selection.

### 6.3 Lifetime of the guide rails

Combining Eqs (9)–(18) and using diameters *D* = 50 mm and *d* = 30mm, we estimated the guide rails service life *t* = 54.2 years.

### 6.4 Measurement of the tension in guide rails

According to Section 6.2 the evaluated critical gangues diameter was 50 mm. The detailed processing and transportation process are as follows: the washed gangues are loaded to the outdoor feeding hopper by the loading machine, then delivered to the screening machine by the belt conveyor. After the screening, gangues with a diameter smaller than 50 mm go directly to the belt, while those with a diameter larger than 50 mm are delivered to the crusher. The crushed gangues which now have diameters less than 50 mm go to the belt. The screened and crushed gangues then transported from the surface to feeding shaft bottoms via the feeding pipe. The speed of these gangues decreased significantly by interacting with the suspended buffer. Since its first operation on December 2014, the suspended buffer’ guide rails have been operating steadily and no fatigue damage was observed.

**Fig 11:**
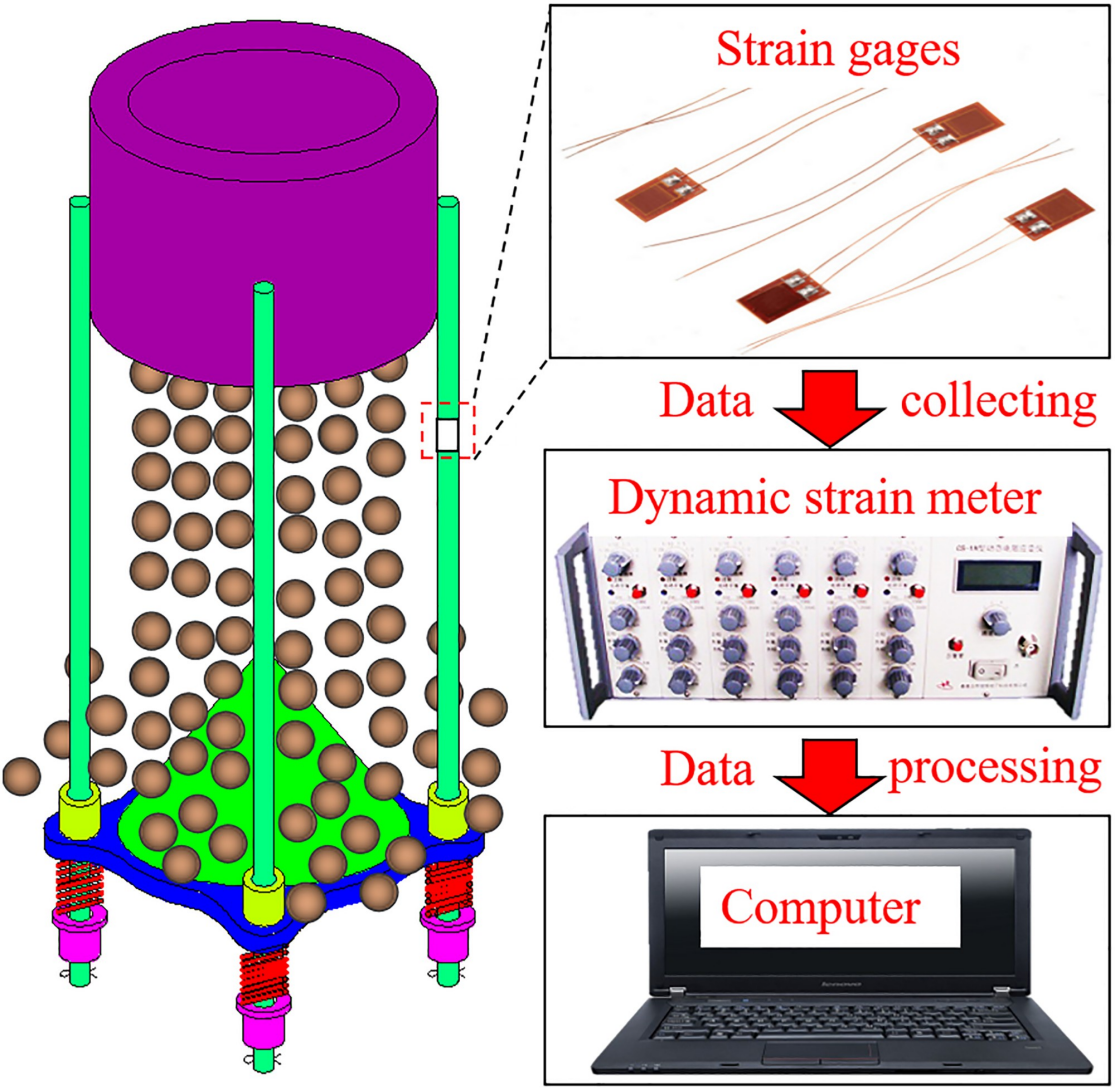
Monitoring scheme.

**Fig 12:**
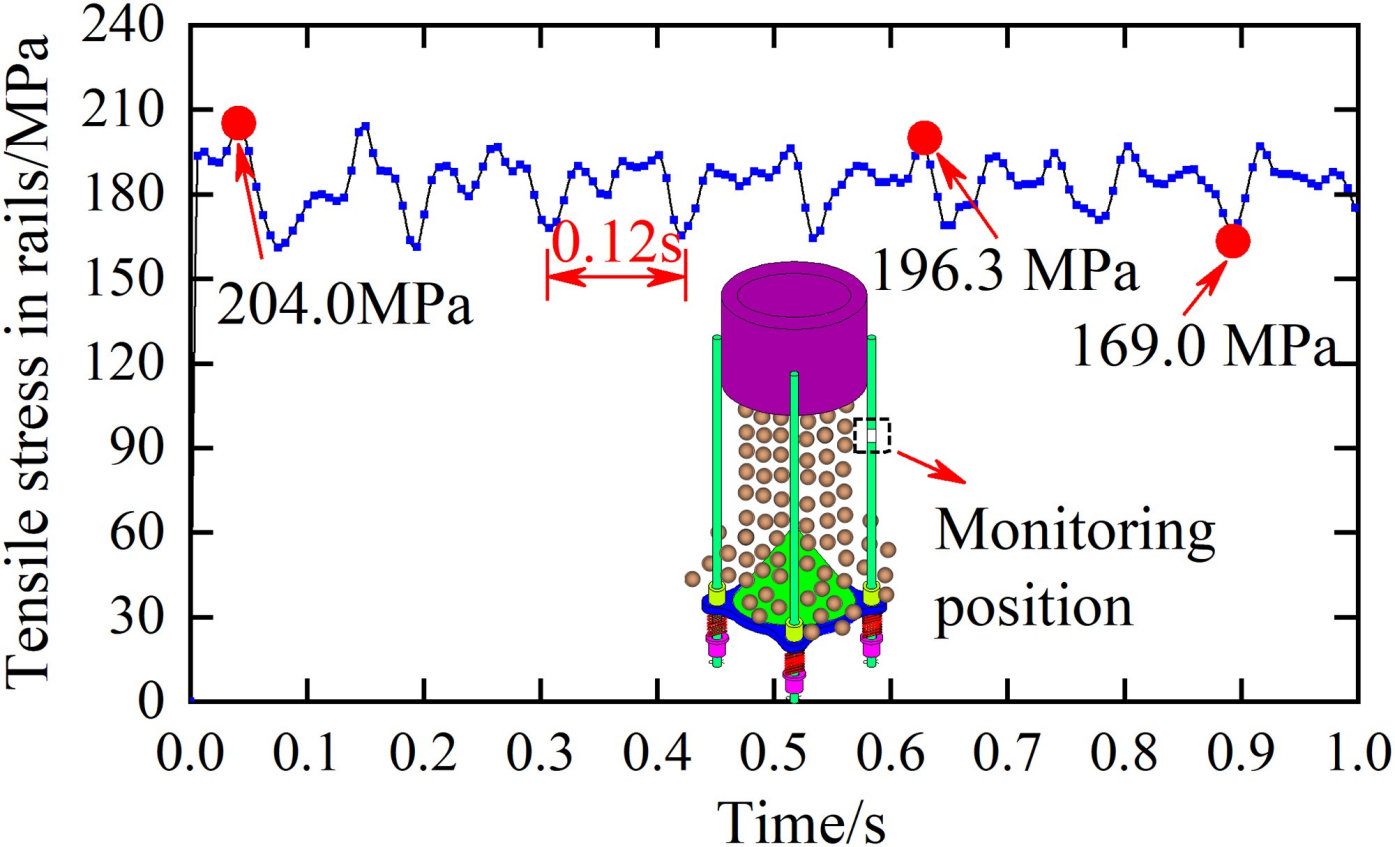
Transient variation of the tensile stress in guide rails.

To further investigate the variation of tension in guide rails due to the collision of gangues with the suspended buffer, strain gages have been placed on the guide rails as shown in Fig 11. The dynamic strain meter was used to obtain the strain, which after the computer processing yielded the tensile stress in rails. Fig 12 shows the transient variation of tensile stress in rails.

Fig 12 shows that the peak tensile stress on the guide rail was *σ*_1_ = 204.2 MPa. Also, the vibration frequency *f* was 8.3 Hz and the maximum and minimum tensile stresses within the vibration period were *σ*_2_ = 196.3 MPa and *σ*_3_ = 169.0 MPa, respectively. To verify the reliability of the numerical simulation, we compared the measured vibration parameters of the guide rails with those predicted by the numerical model. Substituting *v* = 500 t/h and *D* = 50 mm into Eqs (7) and (11)–(13), the model predicted that *σ*_1_ = 224.8 MPa, *σ*_2_ = 221.2 MPa, *σ*_3_ = 183.7 MPa and *f* = 8.5 Hz. Comparing with the measured values, one can obtain that the relative errors for *σ*_1_, *σ*_2_, *σ*_3_, and *f* are 9.3%, 11.3%, 8.0% and 2.4%, respectively. The agreement between the predicted and measured vibration parameters of guide rails verifies the reliability of the simulation to some extent.

## 7 Conclusions

Exploring the structural components of the suspended buffer and design principles of guide rails we developed a numerical vibration model for the suspended buffer by combining the Pro/E and Femap software. The effects of the gangues diameter and the feeding rate on the dynamic response of the buffer’s guide rails were investigated and the following conclusions were drawn:

1. After being impacted by the gangues, the peak tension on guide rails increased rapidly, followed by a periodic vibration.
2. Peak tension, maximum and minimum tensions within the vibration period are exponential functions of the gangues diameter and linear functions of the feeding rate. The vibration frequency does not depend on the feeding rate but depends exponentially on the gangues diameter.
3. The feeding rate for 15601 working panel at D.Ping coal mine is determined to be 500 t/h based on its backfilling workface parameters. The critical gangues diameter of 50 mm was evaluated according to the strength of the material used for the guide rails, which gave an estimated service life of 54.2 years for the rails. Finally, the suspended buffer was tested in D.Ping mine’s 15601 working panel and the guide rails operated steadily in the field. The predicted and measured vibration parameters for the rails agree with each other, that verifies the reliability of the numerical simulation to some extent.
4. By means of numerical simulation, the effect of gangue diameter and gangue feeding speed on the safety and lifetime of guide rails were studied, which provides a basis for the selection of gangue diameter and prediction of lifetime of guide rails. However, the article does not analyze the influence of gangue gradation on the guide rails. This is also the next step of the article.

## References

[1] Yi XW, Ma GW, Fourie A. Centrifuge model studies on the stability of fibre-reinforced cemented paste backfill stopes. Geotextiles and Geomembranes [J]. 2018;46(4):396–401. 10.1016/j.geotexmem.2018.03.004

[2] Yilmaz T, Ercikdi B, Deveci H. Utilisation of construction and demolition waste as cemented paste backfill material for underground mine openings. Journal of environmental management [J]. 2018;222:250–259. 10.1016/j.jenvman.2018.05.075 29859465

[3] Madzivire G, Ramasenya K, Tlowana S, Coetzee H, Vadapalli VRK. Application of mine water leaching protocol on coal fly ash to assess leaching characteristics for suitability as a mine backfill material. Journal of Environmental Science and Health Part a-Toxic/Hazardous Substances & Environmental Engineering [J]. 2018;53(5):467–474. 10.1080/10934529.2017.1410420 29232163

[4] Vargas T, Rojas F, Bahamondez C, Castro R, Ihle CF, Caraballo M, et al Physical and chemical transformations of gangue materials during leaching of copper sulphides, and their influence on copper leaching kinetics. Journal of the Southern African Institute of Mining and Metallurgy [J]. 2017;117(8):727–730. 10.17159/2411-9717/2017/v117n8a1

[5] Zhang JX, Li M, Liu Z, Zhou N. Fractal characteristics of crushed particles of coal gangue under compaction. Powder Technology [J]. 2017;305(1):12–18. 10.1016/j.powtec.2016.09.049

[6] Ju F, Li B, Guo S, Xiao M. Dynamic characteristics of gangues during vertical feeding in solid backfill mining: a case study of the Wugou coal mine in China. Environmental Earth Sciences [J]. 2016;75(20):1389–1389. 10.1007/s12665-016-6194-0

[7] Emad MZ, Mitri H, Kelly C. Dynamic model validation using blast vibration monitoring in mine backfill. International Journal of Rock Mechanics and Mining Sciences [J]. 2018;107:48–54. 10.1016/j.ijrmms.2018.04.047

[8] Zhu WB, Xu JM, Xu JL, Chen DY, Shi JX. Pier-column backfill mining technology for controlling surface subsidence. International Journal of Rock Mechanics and Mining Sciences [J]. 2017;96:58–65. 10.1016/j.ijrmms.2017.04.014

[9] Zhang JX, An BF, JU F, Jiang H-q, Wu Q. Influence Factors of Solid Material Particles Motion in the Feeding System of Fully Mechanized Coal Mining. Journal of Mining & Safety Engineering [J]. 2012;29(3):312–316.

[10] Liu Z, Zhnag JX, Ju F. Vibration and impact analysis of buffer device of vertical material feeding system in solid backfilling coal mining. Journal of Mining & Safety Engineering [J]. 2014;31(2):310–314.

[11] Huang CX, Zhu Q, Li CC, Lin W, Xue DJ. Effects of Micronized Fibers on the Cushion Properties of Foam Buffer Package Materials. Bioresources [J]. 2014;9(4):5940–5950.

[12] Wen WC, Li YX, Fu DM. Study on Impact Energy Absorbing Performance of EPS Buffer Layer of Motorcycle Helmet International Conference on Mechanical & Automation Engineering [J]. 2013;415 (6): 105–109

[13] Wang ZG, Lu ZJ, Tian HQ, Yao S, Zhou W. Theoretical assessment methodology on axial compressed hexagonal honeycomb’s energy absorption capability. Mechanics of Advanced Materials and Structures [J]. 2016;23(6):503–512.

[14] Uddin MS, Shafie NA, Zivkovic G, Iop. Hexagonal Hollow Tube Based Energy Absorbing Crash Buffers for Roadside Fixed Objects. Materials Science & Engineering Conference Series [J]. 2017; 84 (1):15–21.

[15] Ahn K, Huh H, Kwon TS. Development of Buffer Stopper Using Progressive Compression Process for Crash Energy Absorption of a Railway Vehicle. Key Engineering Materials [J]. 2013; 535(1): 365–368

[16] Tian ZF, Tu JY, Yeoh GH. Numerical modelling and validation of gas-particle flow in an in-line tube bank. Computers & Chemical Engineering [J]. 2007;31(9):1064–1072. 10.1016/j.compchemeng.2006.09.008

[17] Kartushinskii AI, Michaelides EE, Rudi YA. Numerical Modeling of Gas-Particle Flows in Vertical Pipes and the Particle Collision Effect. Fluid Dynamics [J]. 2004;39(5):748–755. 10.1007/s10697-005-0008-5

[18] El-Behery SM, El-Askary WA, Hamed MH, Ibrahim KA. Numerical and experimental studies of heat transfer in particle-laden gas flows through a vertical riser. International Journal of Heat and Fluid Flow [J]. 2012;33(1):118–30.

[19] Oanta EM, Panait C, Raicu A. Original Data Preprocessor for Femap/Nastran. Advanced Topics in Optoelectronics, Microelectronics, and Nanotechnologies [J]. 2016; 10(1):10–14.

[20] Zeng H, Gao Q, Zhang W-g, Li Y. The Modal Analysis for Spiral bevel Gear Based on Patran/Nastran. Advanced Materials Research [J]. 2016; 299 (2):1083–1086

[21] Zhao W, Li Y, Wang S. Integrated Simulation and Structure Optimization of Rigid-Flexible Coupling Mechanical Leg Based on PATRAN, NASTRAN and ADAMS. Applied Mechanics & Materials [J]. 2014;614(1):16–18

[22] Shan G, Zhang H, Wang Y, Zeng C. Finite Element Analysis of Elastic Membrane Coupling Based on MSC.patran. Advanced Materials Research [J]. 2011; 308 (11): 1961–1965

[23] LV Y, LV GZ, Zhao QL. Development and Application of RVE Library Based on MSC.PATRAN Platform. Bao HG, Jiang Y, editors2008. pp: 50–55.

[24] Consoli NC, Lopes LD, Consoli BS, Festugato L, Di Sante M, Fratalocchi E, et al Mohr-Coulomb failure envelopes of lime-treated soils. Geotechnique [J]. 2015;65(10):866–868.

[25] Galindo RA, Serrano A, Olalla C. Ultimate bearing capacity of rock masses based on modified Mohr-Coulomb strength criterion. International Journal of Rock Mechanics and Mining Sciences[J]. 2017;93:215–225.

[26] Abeyrathna PAMMB, Abeysiriwardhana WASP, Amarasinghe SW, Ariyasinghe WMSL, Abeykoon AMHS. Simulation on Active Vibration Suppression Using Virtual Spring-Damper Combination. International Conference on Circuits. 2014;115 (6): 1–6

[27] Barry OR, Oguamanam DCD, Zu JW. Nonlinear vibration of an axially loaded beam carrying multiple mass-spring-damper systems. Nonlinear Dynamics [J]. 2014;77(4):1597–1608. 10.1007/s11071-014-1402-5

[28] Lu Z, Yang YL, Lu XL, Liu CQ. Preliminary Study on the Damping Effect of a Lateral Damping Buffer under a Debris Flow Load. Applied Sciences-Basel [J]. 2017;7(2): 201–201.

[29] Wang H, Li W, Bai H, Xiao H, Yu H. Failure Analysis of Q345 Steel Structures on Port Cranes. Applied Mechanics and Materials [J]. 2013; 401(11): 844–847

[30] Zhao X, Zhao J. Experimental Study on Ultra-high Cycle Fatigue Property of Q345 Welded Joint. Advanced Materials Research [J]. 2012;538(3):1488–1491

